# Impact of protein O-GlcNAcylation on neural tube malformation in diabetic embryopathy

**DOI:** 10.1038/s41598-017-11655-6

**Published:** 2017-09-11

**Authors:** Gyuyoup Kim, Lixue Cao, E. Albert Reece, Zhiyong Zhao

**Affiliations:** 1 Department of Obstetrics, Gynecology and Reproductive Sciences,University of Maryland School of Medicine, Baltimore, Maryland USA; 2 Department of Biochemistry and Molecular Biology, University of Maryland School of Medicine, Baltimore, Maryland USA

## Abstract

Diabetes mellitus in early pregnancy can cause neural tube defects (NTDs) in embryos by perturbing protein activity, causing cellular stress, and increasing programmed cell death (apoptosis) in the tissues required for neurulation. Hyperglycemia augments a branch pathway in glycolysis, the hexosamine biosynthetic pathway (HBP), to increase uridine diphosphate-N-acetylglucosamine (UDP-GlcNAc). GlcNAc can be added to proteins by O-GlcNAc transferase (OGT) to regulate protein activity. In the embryos of diabetic mice, OGT is highly activated in association with increases in global protein O-GlcNAcylation. In neural stem cells *in vitro*, high glucose elevates O-GlcNAcylation and reactive oxygen species, but the elevations can be suppressed by an OGT inhibitor. Inhibition of OGT in diabetic pregnant mice *in vivo* decreases NTD rate in the embryos. This effect is associated with reduction in global O-GlcNAcylation, alleviation of intracellular stress, and decreases in apoptosis in the embryos. These suggest that OGT plays an important role in diabetic embryopathy via increasing protein O-GlcNAcylation, and that inhibiting OGT could be a candidate approach to prevent birth defects in diabetic pregnancies.

## Introduction

Congenital birth defects caused by maternal diabetes mellitus (DM) in early pregnancy are complications known as diabetic embryopathy^1, 2^. Malformations of the central nervous system, including exencephaly and spina bifida, are the results of incomplete closure of the neural tube during early embryogenesis, and, thus, are collectively referred to as neural tube defects (NTDs)^3^. In diabetic embryopathy, maternal hyperglycemia perturbs intracellular signaling and function of organelles, including the endoplasmic reticulum (ER) and mitochondria. Dysfunction of the ER results in abnormal protein folding and subsequent ER stress^4, 5^. Perturbation of mitochondrial activity leads to over-generation of reactive oxygen species (ROS) and oxidative stress in cells^6, 7^. Together, these cellular stress conditions induce excessive programmed cell death (apoptosis) in the neural epithelium, resulting in failure of neurulation^2^.

**Figure 1:**
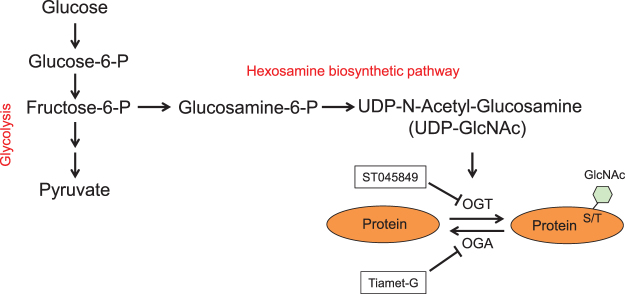
Diagrammatic illustration of the hexosamine biosynthetic pathway (HBP) and O-GlcNAcylation. GlcNAc, generated from HBP, is added to a protein on serine (S) and threonine (T) residues by O-GlcNAc transferase (OGT). This process can be inhibited by ST045849. GlcNAc can be removed from the protein by O-GlcNAcase (OGA), which can be inhibited by Tiamet-G. P, phosphate. UDP, uridine diphosphate.

Glucose within a cell undergoes glycolysis, which produces a number of metabolites, in addition to energy-generating pyruvate. A branch pathway of glycolysis, known as hexosamine biosynthetic pathway (HBP), utilizes a small fraction (~3%) of fructose-6-phosphate to produce uridine diphosphate-N-acetylglucosamine (UDP-GlcNAc; Fig. 1)^8, 9^. GlcNAc can be added to proteins on serine or threonine residues via O-glycosidic linkage. This reaction, known as O-GlcNAcylation, is catalyzed by O-GlcNAc transferase (OGT; Fig. 1)^10^. The reverse reaction is catalyzed by β-N-acetylglucosaminidase or O-GlcNAcase (OGA; Fig. 1)^11^. Thus, O-GlcNAcylation competes with phosphorylation for the same residues to regulate protein function and a wide spectrum of cellular signaling and biological activities^12–15^. O-GlcNAcylation can be modulated at very low concentrations of UDP-GlcNAc (Km 545 nM)^16^. Therefore, HBP is a highly sensitive response to metabolic changes^17^. Under hyperglycemic conditions, glucose influx into cells accelerates intracellular glucose metabolism, an, thus, potentially enhances the HBP pathway and augments protein O-GlcNAcylation^18, 19^.

In diabetic pregnancy, high glucose enters embryonic cells through glucose transporters, enhances intracellular glycolysis, and stimulates multiple glucose metabolic pathways^20–22^. Changes in OGT and OGA have been observed in murine cumulus oocyte complex cultured in high glucose^23, 24^, suggesting a possible involvement of the HBP pathway in mediating the effects of hyperglycemia on development. However, the role of OGT-facilitated O-GlcNAcylation in diabetic embryopathy remains unknown. Understanding the underlying mechanisms, using diabetic animal models which mimic human diabetic pregnancy, may provide information for developing interventions to prevent birth defects in diabetic pregnancies.

## Results

### Global protein O-GlcNAcylation in the neural tube

**Figure 2:**
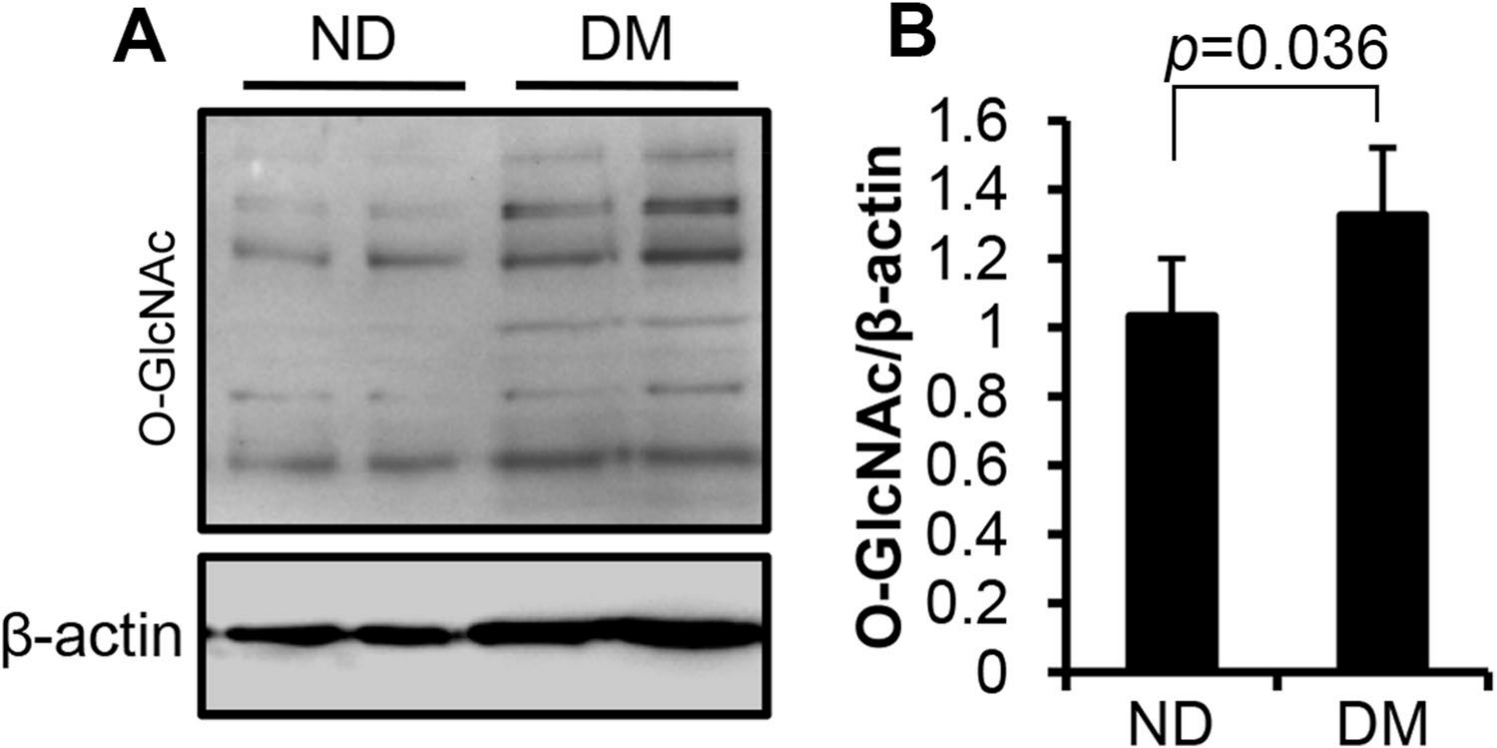
Global protein O-GlcNAcylation in diabetic embryopathy. (**A**) Immunoblot of O-GlcNAcylation in neural tube of E10.5 embryos. β-actin, loading control. (**B**) Quantification of the immunoblot bands. Data are presented as mean ± SD. p = 0.036; n = 5 embryos from 5 litters. DM, diabetic; ND, non-diabetic. Full-length blot of O-GlcNAc is included in the Supplementary Information (Figure S1).

To determine whether the HBP pathway is involved in diabetic embryopathy, we quantified protein O-GlcNAcylation in the neural tube of embryos at E10.5, the late stage of neurulation (E8.5 to E11.5)^25^. The levels of global protein O-GlcNAcylation, measured using immunoblot assay, were significantly increased in the diabetic (DM) group, compared with non-diabetic (ND) group (Fig. 2; *p* = 0.036, n = 5).

### OGT activation in the neural tube

**Figure 3:**
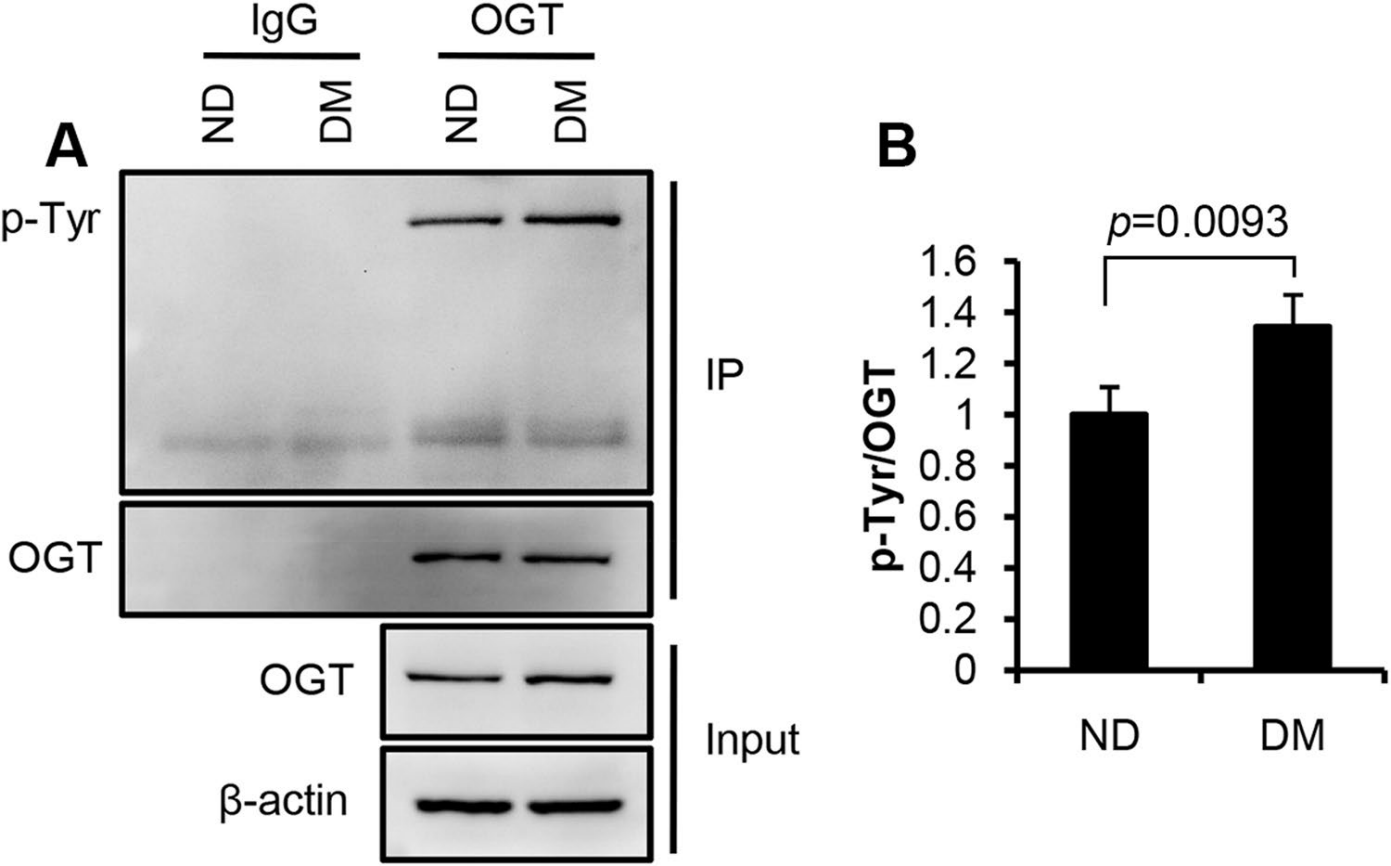
Activation of OGT in the neural tube of embryos from diabetic mice. (**A**) Immunoblot assay of phosphorylated tyrosine residues (p-Tyr) in immunoprecipitated (IP) OGT and total OGT in tissue lysates (input). β-actin, loading control. (**B**) Quantification of the immunoblot bands. Data are presented as mean ± SD. p = 0.0093; n = 3 embryos from 3 litters. DM, diabetic; ND, non-diabetic. Full-length blots are included in the Supplementary Information (Figure S2).

Protein O-GlcNAcylation is regulated by OGT and OGA. We examined the expression of OGT and OGA in the neural tube. No significant changes were observed between the ND and DM groups (Fig. 3; data of OGA not shown). To determine whether the elevation of O-GlcNAcylation is a result of OGT activation, we examined the levels of activated form of OGT, i.e., phosphorylated at tyrosine residues^26^. OGT was immunoprecipitated from the neural tissues. The ratio of phosphorylated OGT in total OGT in the DM group was significantly higher than that in the ND group (Fig. 3; p = 0.0093).

### Effect of high glucose on the increases in O-GlcNAcylation

**Figure 4:**
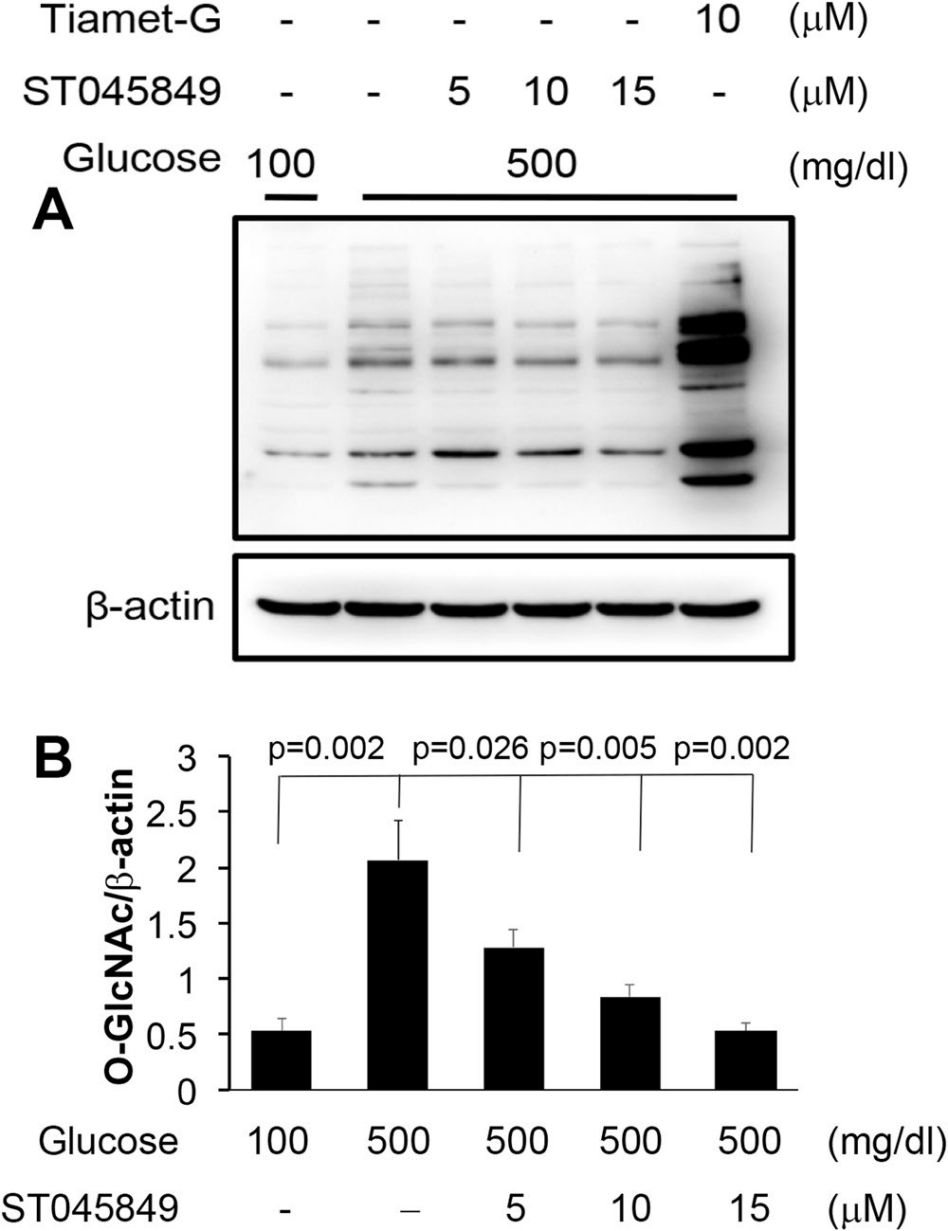
Effect of high glucose on protein O-GlcNAcylation in neural stem cells. (**A**) Immunoblot of O-GlcNAcylation in cell lysates (full-length blot). β-actin, loading control. (**B**) Quantification of the immunoblot bands. Data are presented as mean ± SD. Each treatment is compared with the HG group (Glucose 500). Tiamet-G, OGA inhibitor; ST045849, OGT inhibitor. n = 3 experiments.

To determine whether high glucose increases protein O-GlcNAcylation, we utilized embryonic neural stem cells as a model system^27^. In the cells treated with high glucose (HG; 500 mg/dl), the levels of O-GlcNAcylation were significantly increased (p = 0.002), compared with O-GlcNAcylation levels in cells cultured in normal glucose (NG; 100 mg/dl; Fig. 4).

The increase in O-GlcNAcylation was significantly suppressed by OGT inhibitor ST045849 in a concentration-dependent manner, compared with cells of the HG group (p < 0.05, Fig. 4). Blocking OGA with Tiamet-G resulted in high levels of O-GlcNAcylated proteins, indicating that O-GlcNAcylation was active in the neural stem cells (Fig. 4).

### O-GlcNAcylation in ROS generation

**Figure 5:**
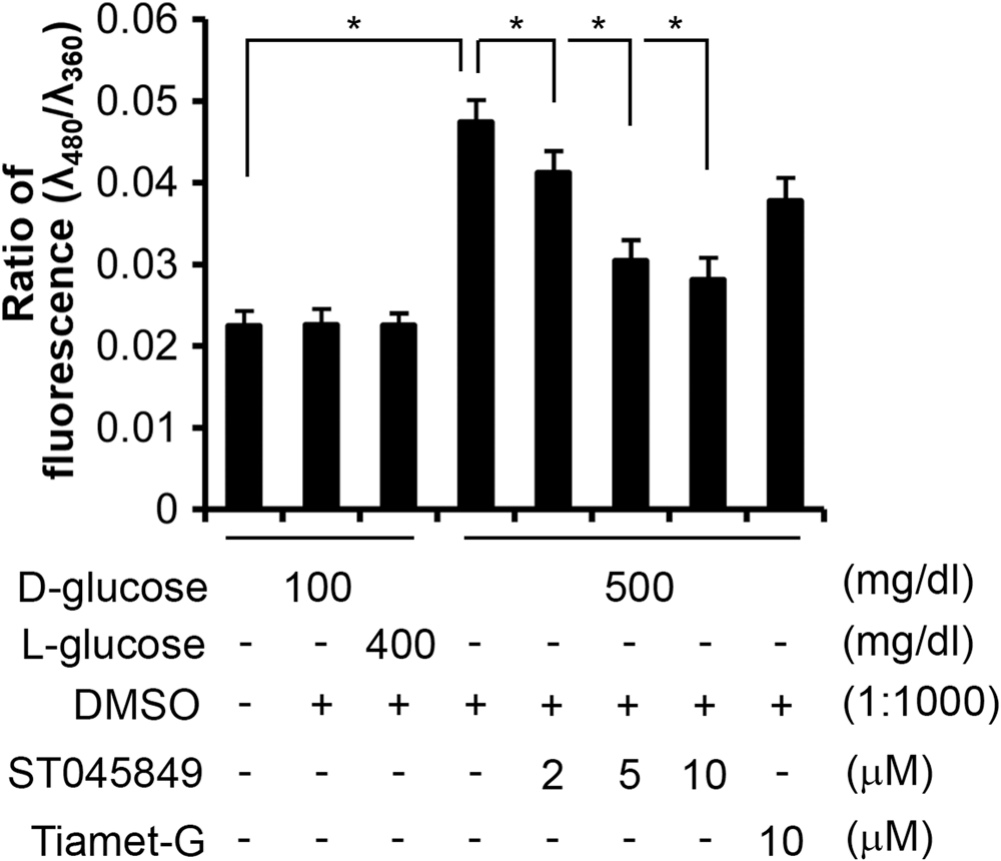
Effect of OGT inhibition on ROS generation in neural stem cells. Cells were treated with high glucose and OGT inhibitor ST045849 or OGA inhibitor Tiamet-G. ROS level is expressed as ratio of fluorescent values between H_2_DCFDA (480 nm) and Hoechst 33342 (360 nm). No difference in the ratio of fluorescent values between Hoechst and propidium iodide (540 nm) was seen among these groups (data not shown). Data are presented as mean ± SD. Each treatment is compared with the group of HG-DMSO group (D-Glucose 500/DMSO). *p < 0.0005; n = 3 experiments. Each treatment in an experiment had eight duplicates. The average values were subject to analysis.

We examined whether increased O-GlcNAcylation is associated mitochondrial function to increase ROS generation in neural stem cells. The levels of ROS in the HG group were significantly elevated, compared with those in the NG group (p < 0.0005; Fig. 5). Blocking OGT activity with ST045849 significantly reduced ROS levels in the cells cultured in HG in a dose-dependent manner (p < 0.0005; Fig. 5), compared with the HG-DMSO group.

### Effect of OGT inhibition on NTD formation

**Figure 6:**
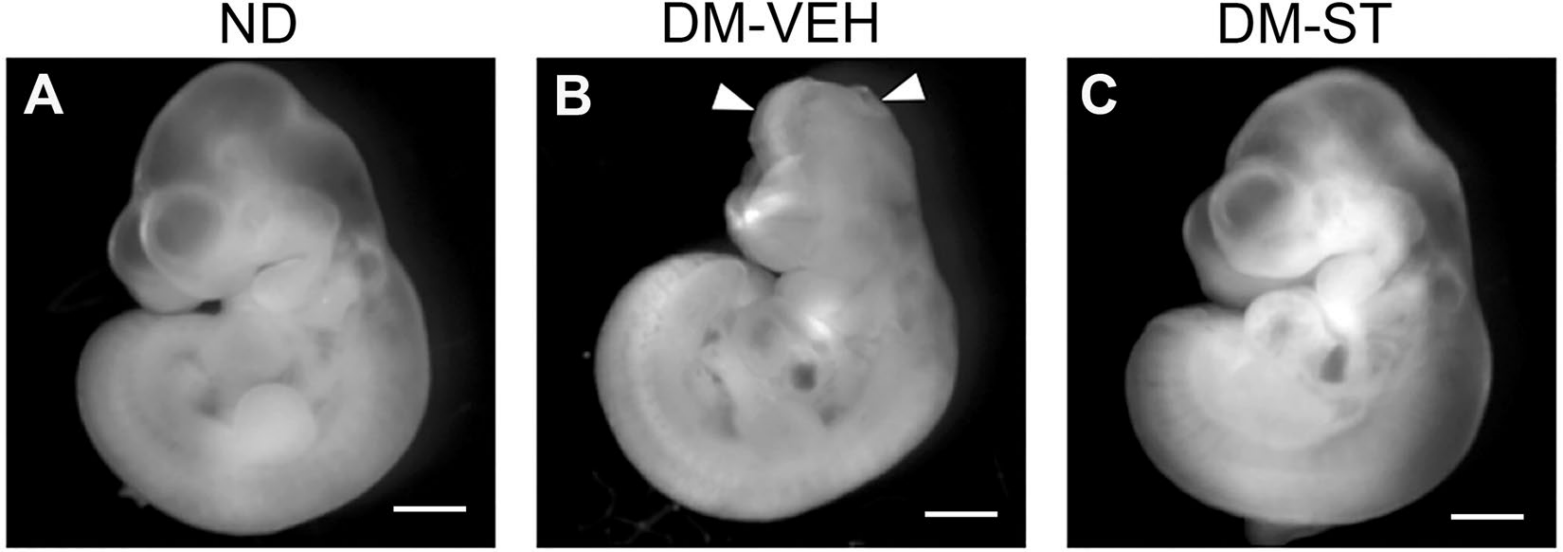
Effect of OGT inhibition on neural tube formation *in vivo*. E10.5 embryos from (**A**) Non-diabetic (ND), (**B**) diabetic and vehicle (DM-VEH), and (**C**) diabetic and ST045849 (DM-ST; 20 mg/kg, daily, from E6.5 to E9.5). Arrowheads indicate open neural tube. Scale bar = 2 mm.

The above experiments showed that hyperglycemia activates OGT and increases O-GlcNAcylation in neural stem cells *in vitro* and the embryos *in vivo*. The next question we wanted to address was whether OGT-catalyzed O-GlcNAcylation plays a role in mediating the effect of maternal hyperglycemia on NTD formation in the embryos. We treated diabetic pregnant mice with ST045849 (20 mg/kg body weight, daily; DM-ST group) from E6.5 to E9.5. NTDs were examined at E10.5 as opened forebrain, midbrain, hindbrain, and/or spinal cord (Fig. 6B). The NTD rate in the diabetic mice treated with vehicle (DM-VEH group) was significantly higher than that in the ND group (Table 1). Treatment with OGT inhibitor (DM-ST group) significantly reduced the NTD rate (1.96%), compared with the DM-VEH group (25.42%; Fig. 6C; Table 1; p = 0.0057).

**Table 1:** Neural tube defect rate in the embryos of diabetic mice treated with OGT inhibitor.

Group	Total Number of Embryos/(litters)	Embryos with NTDs	NTD rate (%)	Blood glucose** (mg/dl)
ND	57/(7)	0	0	183.1 ± 43.5
DM-VEH	59/(7)	15	25.42	341.7 ± 31.6
DM-ST	51/(7)	1	1.96*	380.1 ± 52.7

ND, non-diabetic. DM, diabetes mellitus. ST, ST045849 (OGT inhibitor). NTD, neural tube defect. *p = 0.0057 (DM-VEH vs. DM-ST). **Average values (mean ± SD) from E7.5 to E10.5.

### Effects of OGT inhibition on ER stress and apoptosis

**Figure 7:**
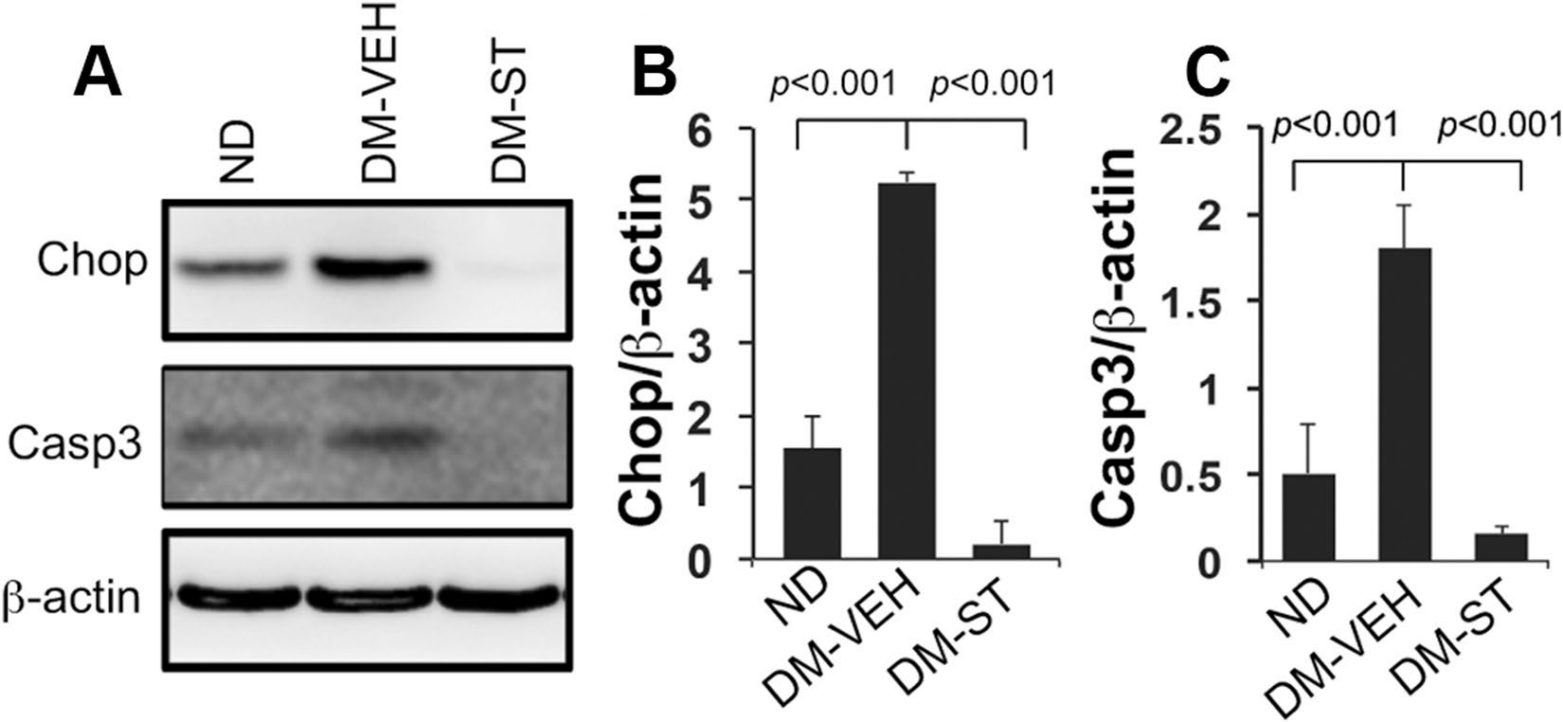
Effects of OGT inhibition on ER stress and apoptosis. Immunoblot assay of protein expression in the neural tissues of E10.5 embryos from non-diabetic (ND), diabetic-vehicle (DM-VEH), and diabetic-ST045849 (DM-ST; 20  mg/kg, daily, from E6.5 to E9.5) groups. (**A**) Immunoblots. β-actin, loading control. (**B**,**C**) Quantification of the immunoblot bands. Data are presented as mean ± SD. n = 3 embryos from 3 litters. Full-length blots of Chop and Casp3 are included in the Supplementary Information (Figure S3).

To investigate the mechanisms underlying the effect of OGT inhibition on reduction of NTDs, we assessed important intracellular factors involved in neurulation in embryos of diabetic dams. OGT inhibition significantly reduced the levels of Chop (C/EBP homologous protein), a biomarker for ER stress, compared with those in the DM-VEH group (p < 0.001; Fig. 7A,B). The levels of apoptosis, indicated by activated (cleaved) Caspase-3 (Casp3), were also decreased in in the DM-ST group, compared with the DM-VEH group, and similar to those in the ND group (p < 0.001; Fig. 7A,C).

## Discussion

GlcNAc is generated during normal glucose metabolism via the HBP pathway and added to proteins post-translationally to regulate protein activity^8, 9^. In hyperglycemia, the HBP pathway is enhanced to generate high levels of GlcNAc and increase protein O-GlcNAcylation. In the embryos of diabetic mice, maternal hyperglycemia activates OGT and increases global protein O-GlcNAcylation. Here we observed that increases in global protein O-GlcNAcylation induce overproduction of ROS and subsequent oxidative stress, as well as ER stress. We found that inhibition of OGT sufficiently reduces the NTD rate, along with ER stress and apoptosis, in the embryos of diabetic dams. These data suggest that OGT plays a role in mediating the effects of hyperglycemia on cellular stress and embryogenesis, and is a potential target for intervention to prevent fetal malformations caused by maternal diabetes in pregnancy.

The HBP pathway, which utilizes only 3% of the glucose in glycolysis, generates the potent cell signaling regulator, GlcNAc, which alters protein and cellular activities via O-GlcNAcylation^28, 29^. As a signaling system sensitive to metabolism and stress, O-GlcNAcylation has been found to be associated with various disorders, including diabetes, neurodegenerative diseases, and cardiovascular diseases^30–33^. In diabetic embryopathy, maternal hyperglycemia can enhance the HBP pathway in embryonic cells with production of GlcNAc. Indeed, GlcNAc levels can be elevated in young embryos cultured in high glucose *in vitro* ^34^. However, the increase in protein O-GlcNAcylation depends on the balance between the activity of OGT and OGA. Our *in vivo* experiments demonstrate the relationship between OGT activation and increases in global protein O-GlcNAcylation.

Alternative splicing of the OGT mRNA generates three isoforms of the enzyme, nucleocytoplasmic OGT (ncOGT), mitochondrial OGT (mOGT), and short OGT (sOGT)^35, 36^. Some ncOGTs are localized in the ER and catalyze O-GlcNAcylation of newly synthesized polypeptides and may influence protein folding^37, 38^. In diabetic embryopathy, hyperglycemia disturbs protein folding. Accumulation of misfolded proteins in the ER lumen generates ER stress, which induces apoptosis in embryonic cells^2^. Our current study demonstrates an important role for OGT in hyperglycemia-induced embryonic malformations; however, its specific role in regulation of protein folding remains to be investigated.

mOGT exerts profound effects on mitochondria by modifying proteins that regulate mitochondrial morphogenesis and function^39–41^. It has been observed that, in the embryos of diabetic pregnancies, maternal hyperglycemia alters the morphology of mitochondria, manifested as fission or fragmentation^6^. Dynamin-related protein 1 (Drp1) plays an essential role in mitochondrial fission. O-GlcNAcylation of Drp1 increases its activity to restrain mitochondrial membranes^42^. Regulation of mitochondrial dynamics and function also involves members of the Bcl-2 family^43, 44^. It has been shown that O-GlcNAcylation influences the activity of pro-apoptotic Bax and Bad, as well as anti-apoptotic Bcl-2^45–47^. These members of the Bcl-2 family are involved in diabetic embryopathy^2^.

Alterations in mitochondrial morphology and activity perturb the normal function of the organelle^48, 49^, including disruption of the electron transport chain to over-generate ROS. Oxidative stress is one of the major stress conditions in the embryos of diabetic pregnancies^2^. Here we show that inhibition of OGT blunts high glucose-induced ROS generation in neural stem cells *in vitro* and alleviates oxidative stress in the embryos *in vivo*, suggesting that O-GlcNAcylation exerts profound effects on mitochondrial function and oxidative stress in diabetic embryopathy.

O-GlcNAcylation competes with phosphorylation on proteins at serine and threonine residues^50^. It has been shown that maternal hyperglycemia also increases protein phosphorylation^2^. Therefore, such competition can alter protein activity in either adverse or beneficial ways for cell survival, depending on the nature of the proteins and signaling systems that they are involved in. Future work is aimed at specifically characterizing the modifications of key cell survival and apoptotic regulators in diabetic embryopathy.

Although the HPB pathway only takes up about 3% of the glucose in glycolysis, its biological effects via protein modification are significant, and under high glucose conditions such effects are more profound. Therefore, suppression of this pathway may mitigate the effects of hyperglycemia on cellular activity. In our study, blocking OGT reduced levels of ROS in neural stem cells cultured in high glucose conditions. More importantly, OGT inhibition ameliorated ER and oxidative stresses and, ultimately, decreased NTD rate in the embryos of diabetic mice. These data demonstrate the candidacy of OGT inhibition as a means of preventing birth defects caused by diabetes in pregnancy. Inhibitors of OGT may, potentially, be used in concert with antioxidants and chemical chaperones to alleviate oxidative and ER stress conditions to achieve full protection of the embryos in diabetic pregnancies.

## Methods

### Diabetic animal model and *in vivo* treatment

A mouse model of diabetic pregnancy, which mimics human diabetic embryopathy, was generated. The use of animals was approved by the Institutional Animal Care and Use Committee of University of Maryland, Baltimore. All experiments were performed in accordance with relevant guidelines and regulations. Ten-week-old C57BL/6J female mice were intravenously injected with streptozotocin (Sigma-Aldrich) in 0.1 mM citrate buffer at 65 mg/kg body weight. Blood glucose levels were measured via tail clipping using Therasense FreeStyle Lite Blood Glucose Monitoring System (Abbott). The values ≥250 mg/dl or 14 mM indicated diabetes Mellitus (DM). Normal glucose levels (~150 mg/dl or ~8 mM) were restored by subcutaneous implantation of insulin pellets (Linshin Canada). A group of sham-operated mice were used as non-diabetic (ND) controls. Female mice were paired with normal male mice in the afternoon. The presence of vaginal plug on the next morning was designated as embryonic (E) day 0.5. Insulin pellets were removed at E5.5 to make the female animals in the DM group hyperglycemic again before neurulation begins at E8.5^25^.

Diabetic pregnant mice were fed via gavage with an OGT inhibitor ST045849 (TimTec; 20 mg/kg body weight, daily)^51, 52^, suspended in water (vehicle or VEH), from E6.5 to E9.5. At E10.5 (late neurulation), the mice were euthanized and the embryos were collected for examination.

### Cell-based ROS assay

Neural stem cells (NE-4C; American Type Culture Collection), derived from the brain of E9 mouse embryos^27^, were plated on 96-well clear bottom culture plates (2 × 10^4^ cells/well) in Dulbecco’s Modified Eagle Medium (DMEM; Life Technologies) supplemented with 10% fetal bovine serum, 6 mM glucose (normal glucose, NG), and antibiotics, in humidified 95% room air/5% CO_2_ at 37 °C.

The cells cultured in a high concentration of glucose (HG; 500 mg/dl) were treated with an OGT inhibitor, ST045849, or vehicle, dimethyl sulfoxide (DMSO; 1:1000), for 6 hours. Treated cells were incubated with fluorescent dyes for 15 minutes at 37 °C, H_2_DCFDA (2′7′-dichlorodihydrofluorescein diacetate; Life Technologies; 5 µM), is a cell membrane permeable dye to measure ROS. Hoechst 33342 (2 µM) and propidium iodide (1 µg/ml) are DNA dyes to detect total and dead cells, respectively. After the cells were washed with FluoroBrite DMEM (Life Technologies) twice, the levels of fluorescence were measured using a microplate reader (Biotek Synergy) at 480 nm (H_2_DCFDA), 360 nm (Hoechst 33342), and 540 nm (propidium iodide). Experiments were repeated three times. Each treatment had eight duplicates in each experiment.

### Immunoprecipitation and immunoblot assays

The neural tubes in the dorsal-anterior region of the brain were isolated from E10.5 embryos in cold phosphate buffered saline (PBS) under a dissection microscope and individually collected. The neural tissues were homogenized in an immunoprecipitation (IP) lysis buffer (150 mM NaCl, 10 mM Tris-HCl; pH 7.4), 1 mM EDTA, 1 mM EGTA, 0.2 mM sodium ortho-vanadate, 0.2 mM PMSF, 1% Triton X-100, 0.5% Nonidet P40, protease inhibitors), or an immunoblotting lysis buffer [25 mM Tris-HCl, pH 7.6, 150 mM NaCl, 1% NP-40, 1% sodium deoxycholate, 0.1% sodium dodecyl sulfate (SDS)] containing protease and phosphatase inhibitors. The homogenates were centrifuged at 14,000 rpm for 15 minutes at 4 °C to obtain supernatants.

IP was performed by an initial incubation of the tissue lysates with Protein A agarose beads (Cell Signaling Technology) for 30 minutes, followed by an incubation of the supernatants with fresh Protein A agarose beads and an anti-OGT antibody (Santa Cruz Biotechnology, Santa Cruz, CA) or IgG controls at 4 °C for 16 hours. After washing three times with the lysis buffer, precipitated proteins were eluted in Laemmli SDS buffer and subjected to immunoblot assay.

Protein samples were resolved in 10% polyacrylamide gel using electrophoresis in presence of SDS and blotted onto polyvinylidene fluoride membranes (Millipore, Billerica, MA). After blocking with 10% non-fat milk or bovine serum albumin, the membranes were incubated with primary antibodies [Chop, cleaved Caspase-3 (Cell Signaling Technology), O-GlcNAc, OGT (F-12), OGA (Santa Cruz Biotechnology), and phosphotyrosine (ThermoFisher; PY20)] for 16 hours at 4 °C, followed by incubation with horseradish peroxidase-conjugated secondary antibodies (Santa Cruz Biotechnology) for 1 hour at room temperature. Signals were detected using SuperSignal West Pico Chemiluminescent Substrate (Thermo Scientific). Images were captured and density of the bands was measured using the UVP Bioimage system (UVP).

The same membranes were stripped using Restore Western Blot Stripping Buffer (Thermo Scientific) and probed again with an antibody against β-actin (Abcam) to control for equal loading of protein samples. The values of β-actin band density were used to normalize those of the corresponding bands of interest.

### Statistical analyses

NTD rate was calculated as a percentage of the embryos with NTDs out of total number of embryos. Log binomial models for clustered data were applied to compare the NTD rates between groups, with calculated confidence intervals. Ratios of band density (protein of interest/β-actin; p-OGT/OGT) and fluorescence intensity at two different wavelengths were presented as Mean ± standard deviation (SD) and analyzed using Student’s *t*-test. A *p*-value of < 0.05 was considered statistically significant.

## Electronic supplementary material

Supplementary information
