# Supplementary material for: Impact of protein O-GlcNAcylation on neural tube malformation in diabetic embryopathy
Source: Sci Rep. 2017 Sep 11;7:11107. doi: 10.1038/s41598-017-11655-6 (PMC5593976; doi:10.1038/s41598-017-11655-6)
Supplement: Supplementary file 1 — Supplementary information [file 41598_2017_11655_MOESM1_ESM.pdf]

Impact of protein O-GlcNAcylation on neural tube malformation in diabetic embryopathy

Gyuyoup Kim[1], Lixue Cao[1], E. Albert Reece[1,2], Zhiyong Zhao[1,2]

[1]Department of Obstetrics, Gynecology and Reproductive Sciences, [2]Department of Biochemistry and Molecular Biology, University of Maryland School of Medicine, Baltimore, Maryland

Supplementary Information

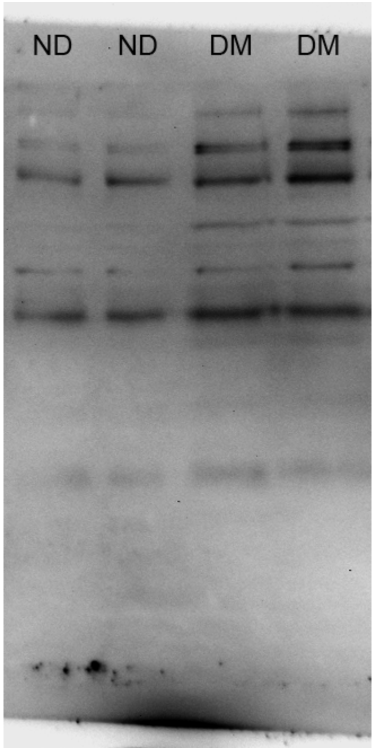

Figure S1. Full-length blot for Figure 2. O-GlcNAcylated proteins in embryonic neural tissues. DM, diabetic; ND, non-diabetic.

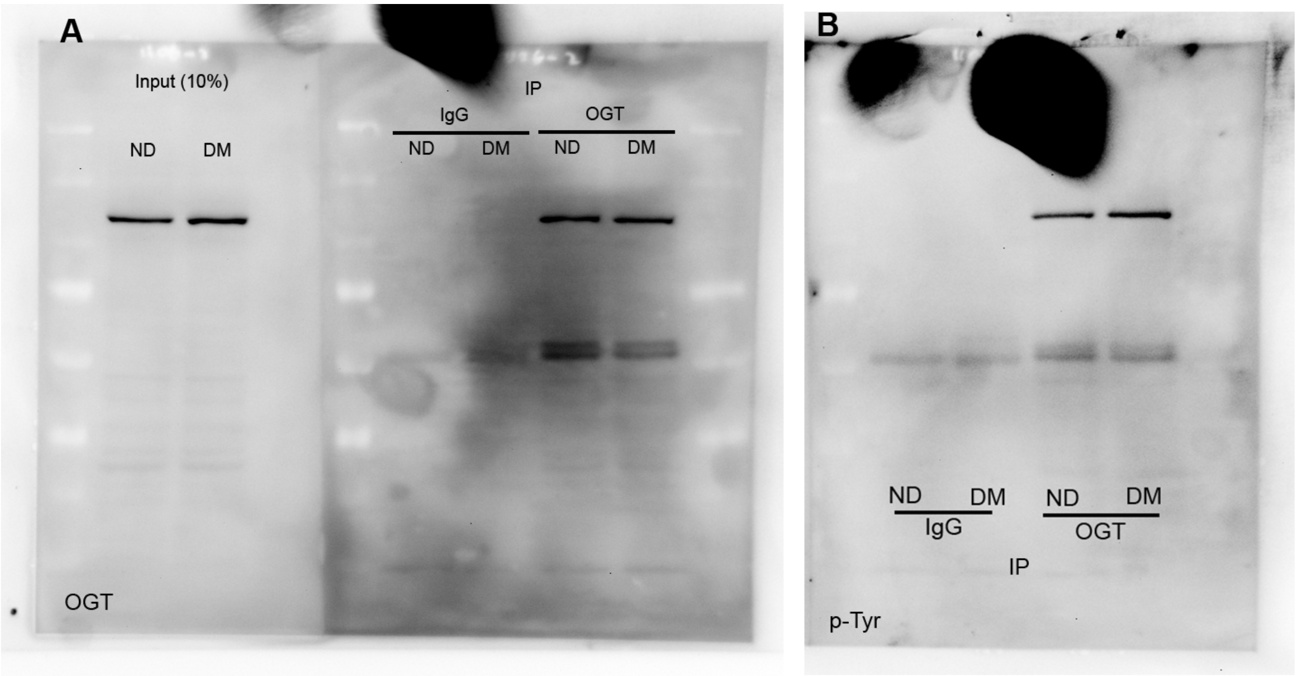

Figure S2. Full-length blots for Figure 3. Immunoprecipitation (IP) of OGT and detection of its phosphorylation at tyrosine residues (p-Tyr). (A) OGT. (B) p-Tyr. DM, diabetic; ND, non-diabetic.

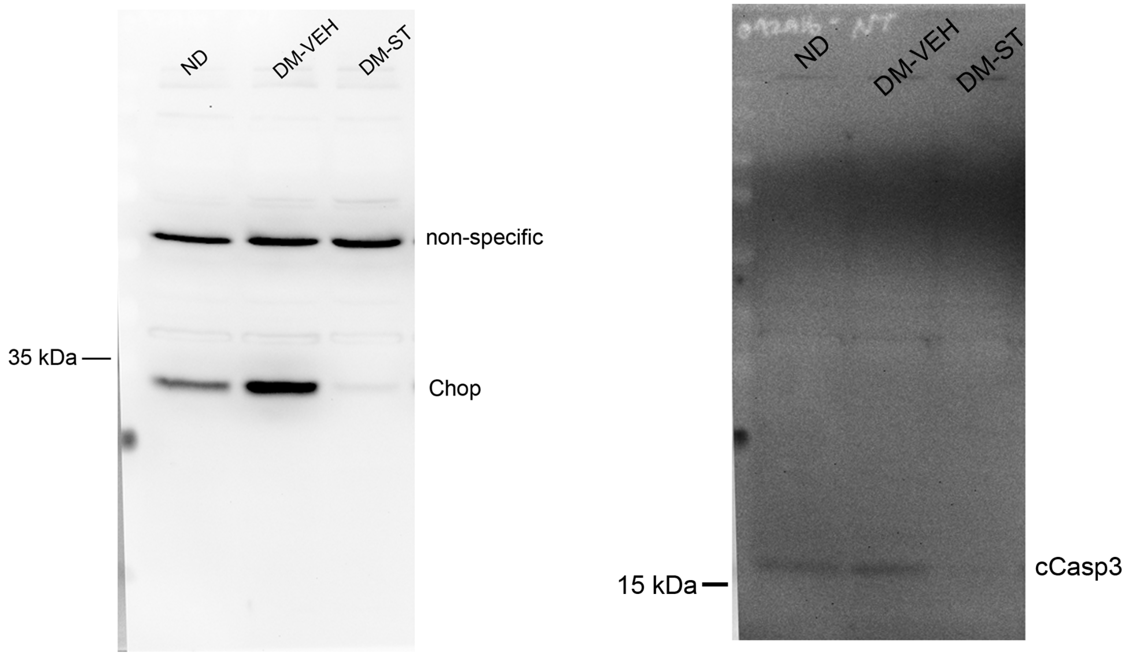

Figure S3. Full-length blots for Figure 7 to show Chop (30 kDa) and cleaved Caspase3 (cCasp3; 18 kDa). DM, diabetic; ND, non-diabetic; VEH, vehicle; ST, ST045849 (OGT inhibitor).
